# Regulation and Physiological Significance of the Nuclear Shape in Plants

**DOI:** 10.3389/fpls.2021.673905

**Published:** 2021-06-10

**Authors:** Chieko Goto, Ikuko Hara-Nishimura, Kentaro Tamura

**Affiliations:** ^1^Graduate School of Science, Kobe University, Kobe, Japan; ^2^Faculty of Science and Engineering, Konan University, Kobe, Japan; ^3^School of Food and Nutritional Sciences, University of Shizuoka, Shizuoka, Japan

**Keywords:** nuclear shape, nuclear envelope, nuclear lamina, nucleoplasmic reticulum, *Arabidopsis thaliana*

## Abstract

The shape of plant nuclei varies among different species, tissues, and cell types. In *Arabidopsis thaliana* seedlings, nuclei in meristems and guard cells are nearly spherical, whereas those of epidermal cells in differentiated tissues are elongated spindle-shaped. The vegetative nuclei in pollen grains are irregularly shaped in angiosperms. In the past few decades, it has been revealed that several nuclear envelope (NE) proteins play the main role in the regulation of the nuclear shape in plants. Some plant NE proteins that regulate nuclear shape are also involved in nuclear or cellular functions, such as nuclear migration, maintenance of chromatin structure, gene expression, calcium and reactive oxygen species signaling, plant growth, reproduction, and plant immunity. The shape of the nucleus has been assessed both by labeling internal components (for instance chromatin) and by labeling membranes, including the NE or endoplasmic reticulum in interphase cells and viral-infected cells of plants. Changes in NE are correlated with the formation of invaginations of the NE, collectively called the nucleoplasmic reticulum. In this review, what is known and what is unknown about nuclear shape determination are presented, and the physiological significance of the control of the nuclear shape in plants is discussed.

## Introduction

The cell nucleus (hereinafter referred to as the nucleus) of eukaryotes consists of chromatin, other nuclear contents (molecules forming/not forming nuclear bodies, such as nucleolus), and surrounding nuclear envelope (NE; Dreger et al., 2001). Usually, the nucleus is observed by labeling chromatin, a nuclear protein, or a NE protein by staining or expressing a fluorescent protein fusion. Although nuclei of most cells are either spherical/round or ellipsoid/oval in shape, alterations of the nuclear shape and size occur, and the alterations are associated with differentiation, aging, and diseases (Webster et al., 2009). Therefore, nuclear shapes can be indicators or diagnostic markers of differentiation stages and pathological states (Zink et al., 2004; Skinner and Johnson, 2017), although the link between the nuclear shape and the physiological function of the nucleus of an organism is unclear.

**Figure 1:**
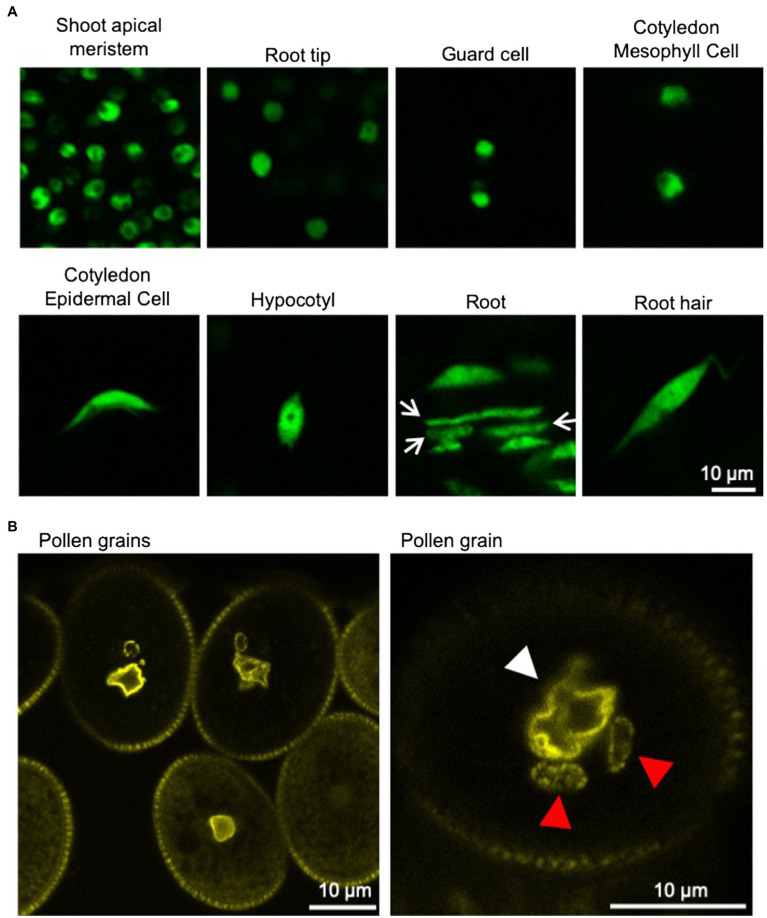
Nuclei in various tissues of *Arabidopsis thaliana*. **(A)** Confocal microscope images of nuclei in various tissues of 6-day-old *A. thaliana* seedlings stably expressing GFP-tagged histone protein (HTA8-GFP) under the control of 35S promoter. Spherical-shaped nuclei are observed in shoot apical meristems, root tips, guard cells, and cotyledon mesophyll cells (upper panels). Spindle-shaped nuclei are observed in cotyledon epidermal cells, hypocotyls, root cells, and root hairs (lower panels). Rod-shaped nuclei (arrow) are also observed in roots. **(B)** Confocal microscope images of nuclei in pollen grains of *A. thaliana* stably expressing YFP-tagged nucleoporin protein (RAE1-EYFP) under the control of 35S promoter (Goto et al., 2020). Fluorescently labeled NE clearly shows the irregular shapes of the VNs (white arrowhead). Sperm cell nuclei (red arrowhead) are often elongated.

Plants have several cell types with different shape, such as polygon-shaped meristematic cells, cylindrical-shaped root cells, and jigsaw puzzle-like-shaped leaf epidermal pavement cells. In meristematic cells, nuclei are small and become larger during differentiation. The nuclei in differentiated cells are often appressed to the plasma membrane by a large vacuole. Regardless of the shape or the differentiation state of the cells, the shape of the nuclei is usually either spherical/round or ellipsoid/oval. However, the vegetative nucleus (VN) of pollen is irregularly shaped and often lobulated in various plant species (Dumas et al., 1985; Heslop-Harrison and Heslop-Harrison, 1989a,b,c; Borges et al., 2012). Additionally, the sperm cell nuclei (SCNs) of plants are often elongated (Borg and Twell, 2011). In the first model plant *Arabidopsis thaliana*, the shape of nuclei in epidermal cells, except for guard cells, changes from spherical to spindle shape during differentiation (Figure 1; Tamura and Hara-Nishimura, 2011; Meier et al., 2016).

Studies of nonplant organisms have identified the factors that influence the nuclear shape, such as NE proteins, cytoskeletons, vesicle trafficking, lipid biosynthesis, chromatin structure, and mitosis. Such NE proteins include lamins, which are intermediate filament proteins that form the scaffold called nuclear lamina (Dechat et al., 2008; Hampoelz and Lecuit, 2011), some lamin-binding proteins, such as lamin B receptor (Ellenberg et al., 1997; Schirmer and Foisner, 2007; Wilson and Foisner, 2010; Hampoelz and Lecuit, 2011), the inner nuclear membrane (INM) protein in *Drosophila* called Kugelkern (Brandt et al., 2006; Polychronidou et al., 2010), other INM proteins called Sad1/UNC-84 (SUN) domain proteins (Starr and Fridolfsson, 2010), outer nuclear membrane (ONM) proteins called Klarsicht/ANC-1/Syne/homology (KASH) domain proteins (Starr and Fridolfsson, 2010), and the nucleoporin Nup153 (Zhou and Panté, 2010). Whereas the actin cytoskeleton pulls nuclei, microtubules (MTs) exert compressive forces on the nucleus in metazoans (Mazumder and Shivashankar, 2010; Hampoelz and Lecuit, 2011). Vesicle trafficking is also reported to maintain the nuclear shape (Kimata et al., 1999; Webster et al., 2009). In yeast, the deletion of certain genes affecting lipid biosynthesis (e.g., *SPO7*, *NEM1*, or *PAH1*) or early protein secretion pathways (*SEC31*, *SEC53*, and *SAR1*) leads to both peripheral ER and NE expansion (Walters et al., 2012). The SWI/SNF chromatin-remodeling enzyme ATPase, BRG1, has been shown to regulate nuclear shape (Imbalzano et al., 2013). Chromatin histone modification and rigidity affect nuclear morphology (Stephens et al., 2018). In *Aspergillus nidulans*, a mitotic NE tether for Gle1 also affects nuclear and nucleolar architecture (Chemudupati et al., 2016).

Plants have no homologous lamin genes (Meier, 2007). Instead, NE proteins that regulate nuclear shape in plants have been identified one after another in the last 15 years (Meier et al., 2016). In addition, it has been suggested that NE proteins or the nuclear shape controlled by them affects nuclear function (migration of the nucleus, chromatin regulation, gene expression, and meiosis) and plant physiological functions (viability, reproduction, plant defense, and salicylic acid response; Evans et al., 2020; Groves et al., 2020). This review introduces what is known and what is unknown about the factors involved in controlling nuclear shape in plants, and discusses about the role of the factors or the nuclear shape in plant physiology.

## Structure and Visualization of the Nucleus

### Structure of the Nucleus

**Figure 2:**
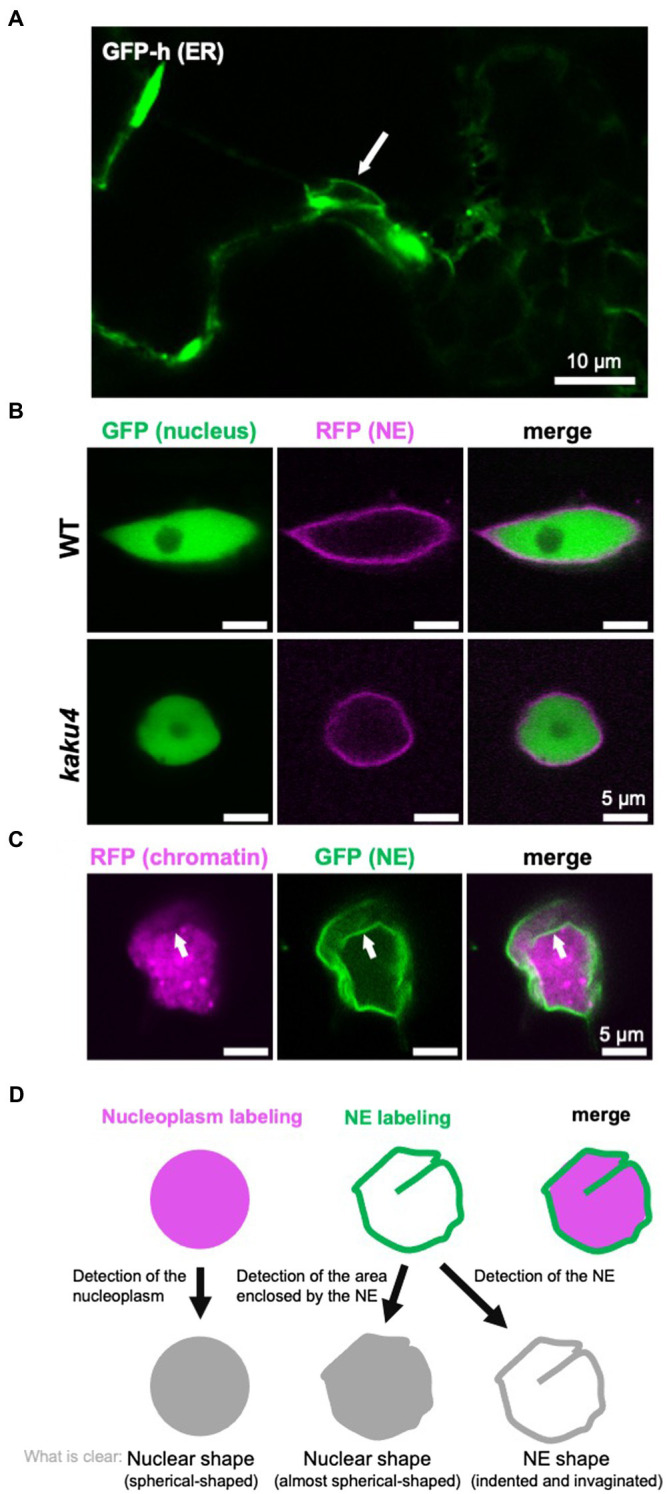
Detection and definition of the nuclear shape and the NE shape. **(A)** A confocal microscope image of *A. thaliana* cotyledon epidermal cells of 7-day-old GFP-h (Mitsuhashi et al., 2000; Hayashi et al., 2001; Nakano et al., 2009), in which ER is fluorescently labeled by GFP. The NE, contiguous with ER, is also visualized. The white arrow indicates a nucleus. **(B)** Confocal images of *A. thaliana* WT and *kaku4* root cells stably coexpressing Nup50a-GFP (Tamura et al., 2010, 2013; Goto et al., 2014) and SUN2-TagRFP (Tamura et al., 2013), each under the control of the 35S promoter. The nucleus, except for the nucleolus, is fluorescently visualized by Nup50a-GFP, and the NE is fluorescently visualized by SUN2-TagRFP. **(C)** Confocal images of *Nicotiana tabacum* leaf cells transiently coexpressing H2B-TagRFP and KAKU4-GFP (Goto et al., 2014), each under the control of the 35S promoter. The chromatin is fluorescently visualized by H2B-GFP, and the NE is fluorescently visualized by KAKU4-GFP. The NE invagination (white arrow) is clearer when the NE is visualized. **(D)** Diagram depicting “nuclear shape” and “NE shape.” In this review, the shapes outlined by both nucleoplasm labeling and NE labeling are called the nuclear shape, whereas shapes outlined by only the labeled NE are called the NE shape.

The nucleus has a structure in which the chromatin and the other components containing nuclear bodies, such as the nucleolus, are surrounded by the NE (Lanctôt et al., 2007). The NE consists of INM, ONM, nuclear lamina, and nuclear pore complexes (NPCs) in metazoans (Dultz and Ellenberg, 2007) and plants (Graumann et al., 2013; Ciska and de la Espina, 2014). The nuclear lamina is a fibrillar meshwork structure underlying the INM, of which the structure is observed in metazoan (Aebi et al., 1986) and plant (Fiserova et al., 2009) cells by electron microscopy. In yeasts, nuclear lamina-like structure is not observed (Cardenas et al., 1990), although two lamina proteins are immunologically identified (Galcheva-Gargova and Stateva, 1988). INM and ONM can be discriminated by transmission electron microscopy (TEM; Starr and Fridolfsson, 2010), and the space between the INM and the ONM is called the perinuclear space (Prunuske and Ullman, 2006). When the NE is observed with a light microscope, INM and ONM can be discriminated between by labeling them with fluorescent markers specific to each membrane (Xu et al., 2007b). INM and ONM are fused to form what is sometimes referred to as the pore membrane (Prunuske and Ullman, 2006), at which an NPC that consists of ~30 nucleoporins is embedded (D’angelo and Hetzer, 2008; Grossman et al., 2012; Tamura and Hara-Nishimura, 2013). ONM is contiguous with the ER (Starr and Fridolfsson, 2010), and a fluorescent protein that localizes in the ER lumen labels both ER and NE (Figure 2A). Although the NE is a type of ER sheet (Schwarz and Blower, 2016), the NE has specific proteins that interact with chromatin (Mattaj, 2004; Zuleger et al., 2011).

The NE breaks down and reassembles during mitosis in metazoans and plants (Boettcher and Barral, 2013; Pradillo et al., 2019). This type of mitosis is called “open” mitosis. In contrast, the NE is intact during “closed” mitosis in budding yeast, in which the spherical metaphase nucleus transforms into an elongated dumbbell shape in anaphase (Boettcher and Barral, 2013). After mitosis, in some cell types, nuclear shape and size change as the cell differentiates (Dauer and Worman, 2009; Tamura and Hara-Nishimura, 2011; Meier et al., 2016).

### Visualization of the Nucleus That Makes Nuclear Shape and NE Shape Recognizable

When cells are observed under a light microscope, nuclei are visible in the bright field without staining regardless of species. However, nuclei are often fluorescently labeled and observed to obtain a clearer image. For fluorescent labeling of nuclei, 4',6-diamidino-2-phenylindole (DAPI; e.g., Collings et al., 2000) and Hoechst 33342 (e.g., Goto et al., 2014) are typically used, which stain chromatin by intercalating into the minor grooves of DNA (Paul and Bhattacharya, 2012). Other DNA-binding reagents can also be used for the visualization of nuclei. In plants, it is effective to fix the cells to allow the staining solution to penetrate the cell wall. As cells are killed by the fixation, a fluorescent protein fusion of chromatin protein [e.g., H2B-green fluorescent protein (GFP)] or nuclear protein (e.g., NLS-GFP) is used to trace the nuclear shape and/or nuclear movement in living cells. Not only the nucleus but also the NE is often labeled by an NE protein fused with a fluorescent protein (e.g., SUN2-GFP). In many cases, the shape and size of the visualized nucleus are very similar between when the nucleus (chromatin or whole nucleus, excluding the NE) is fluorescently labeled and when the NE is fluorescently labeled. However, differences in the labeling targets result in differences in nuclear shape and size, as below: (1) Labeling chromatin cannot visualize the nucleoplasm, except for chromatin, whereas the labeled NE encloses all contents of the nucleus. (2) The nuclear periphery is not always smooth; the NE itself can deform and result in invaginations into the nucleoplasm or nuclear blebs extruded from the nucleus; invaginations of the NE are collectively called the nucleoplasmic reticulum (NR; see section “NE Shapes Under Normal State”). (3) The deformations of the NE are not always accompanied by consistent changes in the shape of the nucleus visualized by labeling the chromatin or nucleoplasm. For example, in the *Arabidopsis* mutant *kaku1* with a mutation in myosin XI-i, nuclear shapes look different depending on the labeling targets (Tamura et al., 2013). The nuclear surface is smooth when the *kaku1-1* nuclei are visualized by the expression of Nup50a-GFP that diffuses throughout the nucleus. In contrast, the *kaku1-1* NE visualized by an NE marker SUN2-TagRFP is irregularly and intricately invaginated, not detected by labeling the nucleoplasm. Other examples of the difference in the appearance of the nucleus due to the difference in the visualization targets (nucleus/NE) are also shown in Figures 2B,C.

The nuclear shape determined by the nucleoplasm and the nuclear shape determined by the NE enclosing nucleoplasm are very similar but strictly not the same, as discussed above. The contents of the nucleus can show morphological changes not revealed by membrane imaging, and vice versa, under light microscope. When focusing on the NE, NE deformations may be classified into two types for convenience. One is a relatively large NE deformation with nucleoplasm deformation. In this type, both NE and nucleoplasm deformations can be detected under both light and electron microscopes. The other is local NE deformation without most of the nucleoplasm deformation. In this type, relatively small NE deformations can be detected under an electron microscope but hardly under a light microscope, and the shape of nucleoplasm changes little regardless of the microscope for detection. The above two types are sometimes not completely distinguishable. However, the classification of NE deformation as above could help understand the mechanism of change in the nuclear shape. For example, the very small and local changes in nuclear shape by the proliferation of the NE are classified into the latter type of NE deformation above, in which “change in NE shape” or “nuclear membrane growth” may be more proper than “change in nuclear shape” to explain the morphological phenotype of the nucleus.

As mentioned above, the nuclear shape can differ depending on labeling targets and microscopes used for observation. In this review, the shapes of chromatin or nucleoplasm and shapes outlined by the NE that are expected to be consistent with the shape of nucleoplasm are tentatively referred to as the “nuclear shape.” In contrast, the overall or local shapes that can be visualized with the labeled NE but hardly with the labeled nucleoplasm are tentatively referred to as the “NE shape” (Figure 2D).

### Quantification of the Nuclear Shape

Nuclear shape and size are sometimes quantified and statistically analyzed. The circularity index and the nucleus area in a cross-sectional image are often calculated to quantify the morphological characterization of the nucleus. In case the nuclear shape is oval, the major and minor axes could be good indexes to quantify the nuclear shape. To quantify the nuclear shape and size more precisely, it is necessary to obtain and analyze a stack of three-dimensional images. NucleusJ, an ImageJ plugin, a useful tool to analyze nuclear morphology and chromatin organization, was released (Poulet et al., 2015) and updated (Dubos et al., 2020). The quantification of the nuclear shape is not only convincingly showing that the nucleus of some mutants is abnormal compared to the wild-type (WT) nucleus but also used to analyze the nuclear mechanical properties (Hampoelz and Lecuit, 2011).

## Nuclear Shape in Plants

### Various Types of Nuclear Shapes in Plants

In many plants, the nuclear shape is spherical/round or ellipsoid/oval. Nuclei in differentiated epidermal cells in leaves of *Nicotiana tabacum* and *Allium cepa* are spherical/round or ellipsoid/oval. In *A. thaliana*, the nuclear shape differs depending on cell or tissue types (nicely depicted in Meier et al., 2016). In 6-day-old *Arabidopsis* seedlings, nuclei are almost spherical in the root meristem, shoot apical meristem, and guard cells, whereas nuclei in the epidermal tissue of other parts in the root, hypocotyl, and cotyledon are spindle-shaped (Figure 1A; Tamura and Hara-Nishimura, 2011; Goto et al., 2014; Meier et al., 2016). Rod-like nuclei were located within vascular tissues (Chytilova et al., 1999). Recently, it was reported that nucleus compression occurs at the organ-meristem boundary (Fal et al., 2021).

The VN of pollen is irregularly shaped and often lobulated throughout various plant species (Dumas et al., 1985; Heslop-Harrison and Heslop-Harrison, 1989a,b,c; Borges et al., 2012). In mature pollen of *Arabidopsis*, the VN is highly invaginated (Figure 1B; Borges et al., 2012; Meier et al., 2016). The VN moves around in the pollen grain while changing its shape and then enters the pollen tube, in which the VN forms an elongated shape (Zhou and Meier, 2014). Additionally, the SCNs of angiosperm are often elongated (Borg and Twell, 2011). Interestingly, the nucleus becomes elongated with coiling during spermatogenesis in bryophytes (Renzaglia and Duckett, 1987). In most bryophytes, sperm nuclei form an elongated shape with slightly sinistral coiling, except for hornworts, of which nuclei are dextral (Renzaglia and Duckett, 1989). In vertebrates, nonround nuclei are observed in neutrophils, neurons, and spermatozoa (Dauer and Worman, 2009; Webster et al., 2009; Skinner and Johnson, 2017). There seems to be a tendency that nuclei form an elongated or irregular shape when the nucleus itself or the cell containing the nucleus migrates through narrow spaces.

### Physiological Contexts of Nuclear Shape in Plants

In *Arabidopsis* seedlings, the amount of DNA increases due to endoreduplication but at the same time changes the nucleus from circular to spindle shape (Jovtchev et al., 2006; Sliwinska et al., 2012). However, endoreduplication or the increase of DNA content does not seem to be the decisive factor in inducing a change in the shape of the nucleus to a spindle shape as the nucleus remains spherical even after endoduplication in *Barbarea stricta* (Jovtchev et al., 2006). The nuclear shape differs depending on cell or tissue types as mentioned above, which suggests that nuclear morphogenesis, in which spherical nuclei change to the bigger spindle-shaped nuclei, seems to occur during cell differentiation (Tamura and Hara-Nishimura, 2011). Nuclei maintain the ability to change their shape even after they accomplish their morphogenesis as seen in roots (Chytilova et al., 2000; Tamura et al., 2013) and pollen grains (Zhou and Meier, 2014), in which nuclei flexibly change their shapes as they migrate. In contrast, nuclei maintain the spindle shape formed by the morphogenesis after their isolation and each treatment of reagent to break down the NE, actin filaments, and MTs (Sakamoto and Takagi, 2013). The formation and maintenance of the nuclear shape in plants seem to be regulated mainly by NE proteins (see also section “Factors Involved in the Regulation of the Nuclear Shape”). The links between the nuclear shape and NE proteins in plants are revealed by a molecular genetic approach using *Arabidopsis*.

## Shape of the NE in Plants

### NE Shapes Under Normal State

The surface of the nucleus is wrinkled rather than smooth. In nuclei of *A. cepa* stained with DAPI, nuclear grooves and invaginations are observed and also confirmed in nuclei labeled by nucleus-targeted GFP (N-GFP) or ER-targeted GFP (ER-GFP; Collings et al., 2000). Nuclear grooves and invaginations are also observed in cultured cells from *N. tabacum* expressing ER-GFP (Collings et al., 2000; Gupton et al., 2006). F-actin is present in nuclear grooves and surrounds the nucleus in cells of *A. cepa* (Collings et al., 2000). Nuclear grooves and invaginations appear to associate preferentially with nucleoli in epidermal cells of *A. cepa* (Collings et al., 2000). Invaginations of the NE, collectively called the NR, have been reported in various normal and abnormal cells from the plant and animal kingdoms (Malhas et al., 2011). The name nucleoplasmic reticulum (NR) was suggested for the convolutions of the NE, including deep, branching invaginations by morphological comparisons with the endoplasmic reticulum (ER). Common to plants and animals, under normal conditions, NE deformation or NR formation appears to be prominent, especially in reproductive cells and surrounding cells. For example, fluorescent labeling of the NE shows that pollen VNs are irregularly shaped, with invagination of the NE, which is an example of the prominent NE deformation (Figure 1B; Zhou and Meier, 2014; Zhou et al., 2015b). It is also observed that only INM invaginates into the nucleus in the meiocyte of *Marsilea vestita* (Sheffield et al., 1979) and placental cells of *Lilium regale* (Singh et al., 1998). Evaginations of only ONM and invaginations of the NE are also observed in microspores of *Podocarpus macrophyllus* (Aldrich and Vasil, 1970). One of the well-studied animal NRs is a “nucleolar channel system” that develops in postovulation human endometrial cells, and the transient presence of the nucleolar channel system has been associated with human fertility (Kittur et al., 2007). The characteristic NE shape in each tissue may imply the physiological role of the NE specific to that tissue.

### Changes in the NE Shape by a Viral Infection

The deformation of the NE or formation of NR is observed under not only normal but also pathological conditions of plants and animals. It is observed under a TEM that many vesicles are formed in the perinuclear space of pea enation mosaic virus-infected plant cells (De Zoeten et al., 1972), which is reminiscent of the primary enveloped virion in the perinuclear cleft of herpesvirus-infected animal cells (Mettenleiter et al., 2006). TEM analysis also reveals that the infection of cowpea severe mosaic virus and bean yellow mosaic virus induces intranuclear inclusions (Carr and Kim, 1983). Not only TEMs but also fluorescence microscopes detect NE deformations induced by a viral infection. Infection of *Nicotiana benthamiana* with *Potato yellow dwarf virus* (PYDV) or *Sonchus yellow net virus* (SYNV) induces the accumulation of membranes within the nuclei (Goodin et al., 2005). The M protein of PYDV can induce the intranuclear accumulation of the INM in the absence of any other viral protein (Bandyopadhyay et al., 2010). A complex containing M protein of SYNV is discovered that appears to bud from the nucleus and that moves on ER membranes (Goodin et al., 2007). This budding of the M protein implies the budding of mature virions from the perinuclear space, of which the mechanism would be akin to the “bud-in, bud-out” envelopment and de-envelopment of herpesvirus particles at the NE (Goodin et al., 2007). It is proposed that herpesviruses have hijacked a nuclear export pathway employed by endogenous RNPs (Speese et al., 2012).

## Factors Involved in the Regulation of the Nuclear Shape

### Overview of Nuclear Shape Determination in Plants

**Figure 3:**
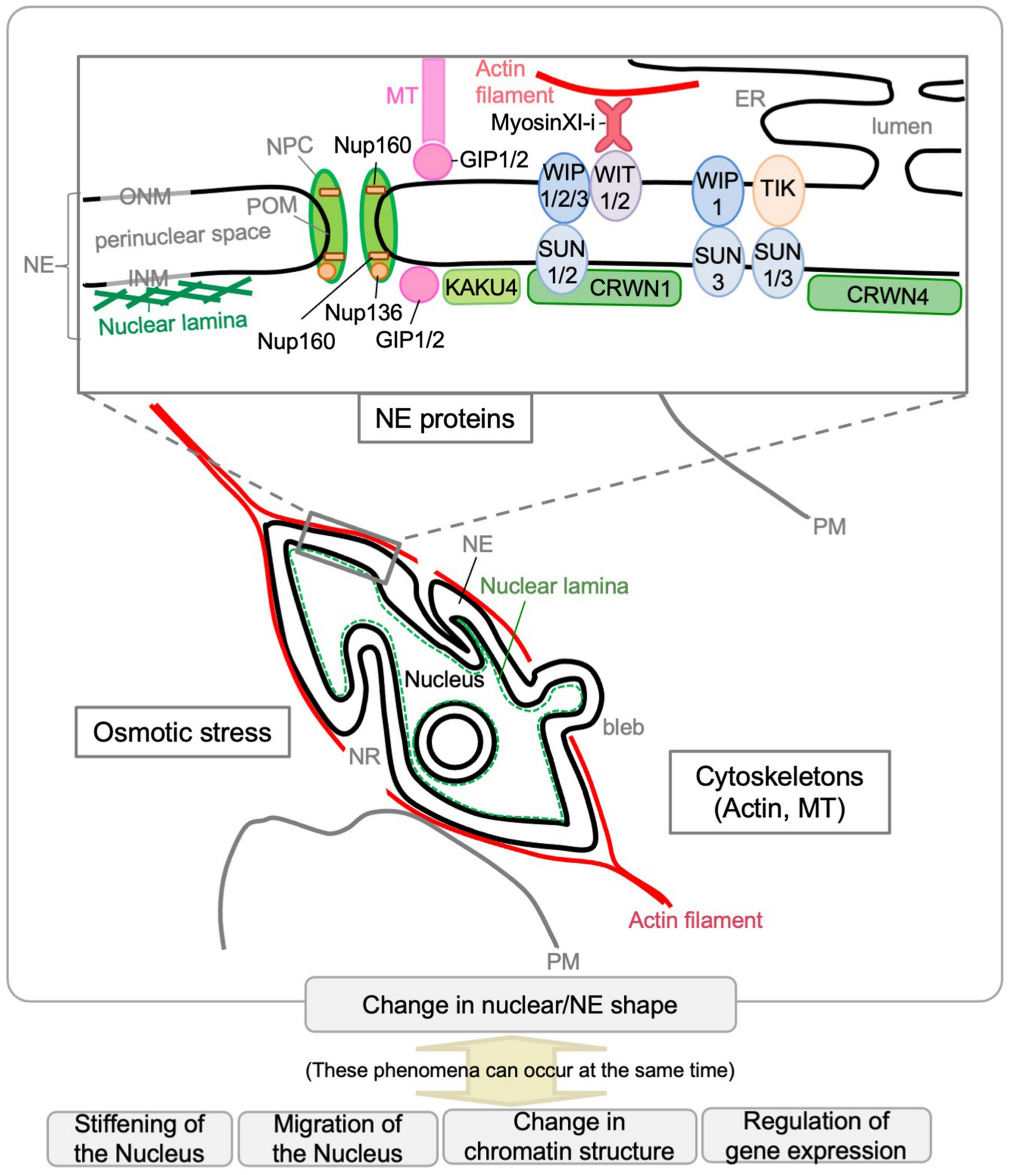
Factors that affect the nuclear shape in plants. Scaffolding or other functions by NE proteins and cytoskeletons (actin and MTs) are involved in controlling the nuclear shape in plants. Osmotic stress affects nuclear shape. The *Arabidopsis* NE proteins that affect nuclear shape are depicted in the enlarged diagram of the NE structure. The phenomena shown at the bottom of the diagram can occur during nuclear shape changes.

Thus far, molecular genetic analyses have revealed that several NE proteins and cytoskeleton-associated proteins are involved in controlling the nuclear shape in plants (Figure 3). Single or multiple mutants deficient in nuclear lamina constituent protein candidates (CRWNs and KAKU4), plant LINC complex components (SUNs, WIPs, and WITs), myosin XI-i, or nucleoporins (Nup136/Nup1 and Nup160) have more spherical nuclei compared with WT. Among them, KAKU4, CRWN4, and Nup136/Nup1 promote NE deformation when overexpressed. Unlike the above, *gip1 gip2* mutant has an abnormally shaped nucleus with jagged edges at the root tip. These findings were summarized by gene in previous reviews (Tamura et al., 2015; Meier et al., 2016). This section briefly reviews the factors involved in controlling nuclear shape after categorization based on their effect on the nuclear shape. It includes recent updates and introduces factors that have been found to control nuclear shapes in animals but with unclear effects on plant nuclear shapes (section “Other Possible Factors That Can Be Involved in Nuclear Shape Determination”). The last part of this section discusses how nuclear shapes in plants are controlled (section “Regulation of the Nuclear Shape by GIP, SUN-WIP-WIT-Myosin XI-i, and CRWN-KAKU4”).

### NE Proteins of Which the Deficiency Results in Small Spherical Nuclei in *Arabidopsis*

Many, but not all, NE proteins are involved in the formation and/or maintenance of nuclear shape (Figure 3). While nuclei become bigger and spindle-shaped during differentiation in WT *A. thaliana*, nuclei remained spherical in the following single or multiple mutants: *crwn1 crwn2*, *crwn1*, *crwn4*, *kaku4*, *sun1 sun2*, *sun3*, *wip1 wip2 wip3*, *wit1 wit2*, *tik*, *myosin XI-i*, *nup136/nup1*, and *nup160*.

#### Candidate Components of the Nuclear Lamina-Like Structure: NMCPs/CRWNs and KAKU4

The *CROWDED NUCLEI1* (*CRWN1*) gene was originally named as *LITTLE NUCLEI1* (*LINC1*; Dittmer et al., 2007), for the *Arabidopsis* homolog of the gene encoding the insoluble protein nuclear matrix constituent protein 1 (NMCP1), isolated from carrots (Masuda et al., 1997). *LINC1* was renamed *CRWN1* in 2013 (Wang et al., 2013). *A. cepa* NMCP1 (Ciska et al., 2013) and *Arabidopsis* CRWN1 (Sakamoto et al., 2020) have been suggested to be localized within the nuclear lamina. The small spherical nuclei in *crwn1 crwn2* (Dittmer et al., 2007) are the first reported nuclei with a shape different to that of the WT. The CRWN family is plant specific, and *Arabidopsis* has three NMCP1 homologs (CRWN1-3) and an NMCP2 homolog (CRWN4; Ciska et al., 2013). Whereas *crwn1* and *crwn4* clearly show spherical nuclei, nuclei in *crwn2* and *crwn3* are less different to those in WT (Sakamoto and Takagi, 2013).

KAKU4 was novelly identified from mutant screening with a focus on nuclear shape (Goto et al., 2014). Mutant screening also resulted in the isolation of *kaku1* (*myosin XI-i*; Tamura et al., 2013) and *kaku2* (another allele of *crwn1*; Goto et al., 2014). KAKU4 is localized near the INM, which suggests its localization to the nuclear lamina (Goto et al., 2014). KAKU4 interacts with CRWN1 and CRWN4 (Goto et al., 2014) and is conserved in seed-bearing plants (Goto et al., 2014; Poulet et al., 2017b). Two *KAKU4*-like genes have been identified in some plant species, including *Glycine max* and *Zea mays* (Poulet et al., 2017b; Gumber et al., 2019a), whereas *A. thaliana* has only one *KAKU4* gene (Goto et al., 2014; Poulet et al., 2017b). Almost all nuclei in *kaku4* seedlings were spherical, similar to *those in crwn1* (Goto et al., 2014). KAKU4 is also required to form irregularly shaped VNs in pollen grains (Goto et al., 2020). Based on their localization and effects on the nuclear shape, CRWN1 and KAKU4 are expected to be functional analogs of the intermediate filament proteins lamin and its binding proteins of metazoans that make up the nuclear lamina (Guo and Fang, 2014; Tamura et al., 2015; Meier et al., 2017; Groves et al., 2020).

#### Components of Plant LINC Complexes and the Myosin: SUNs, WIPs, WITs, Myosin XI-i, TIK, and MLKS2

Analysis of the localization, FRET, and FRAP of the protein fusions suggests that CRWN1 interacts with SUN1 and SUN2 (Graumann, 2014), reminiscent of animal lamins interacting with the INM protein SUNs (Starr and Fridolfsson, 2010). *A. thaliana* SUN1 and SUN2 were identified via a protein BLAST search with the *Caenorhabditis elegans* SUN domain protein UNC-84 sequence as the query (Graumann et al., 2010). Subsequently, a further SUN domain search identified SUN3, SUN4, and SUN5 in *Arabidopsis* (Graumann et al., 2014). These SUNs are classified into two subfamilies based on the position of the SUN domain within the protein: SUN1 and SUN2 are referred to as Cter-SUN, whereas SUN3, SUN4, and SUN5 are referred to as mid-SUN (Graumann et al., 2014). The nuclei of root hairs were rounder in *sun1sun2* than in WT (Oda and Fukuda, 2011; Zhou et al., 2012). Statistical analysis of circularity suggests that *sun3* also has a rounder nucleus compared to the WT (Graumann et al., 2014). The nuclear shape in *sun4* and *sun5* was similar to that in the WT (Graumann et al., 2014).

In metazoans and fungi, SUN domain proteins interact with KASH domain proteins in the perinuclear space to form LINC complexes (Starr and Fridolfsson, 2010). The LINC complex interacts with the nuclear lamina on the nucleoplasmic side and the cytoskeleton on the cytoplasmic side, and is involved in controlling the nuclear shape, migration of the nucleus, and mechanotransduction from the cell membrane to the nucleus (Dahl et al., 2008; Starr and Fridolfsson, 2010). In plants, some components of the LINC complexes are involved in controlling nuclear morphology: *Arabidopsis* SUN-WIP-WIT complex (Zhou et al., 2012; Tamura et al., 2013), *Arabidopsis* SUN3-TIK complex (Graumann et al., 2014), and *Z. mays* SUN2-MLKS2 complex (Gumber et al., 2019b). In *Arabidopsis*, SUN proteins (SUNs) interact with WIP proteins (WIPs), WIPs interact with WIT proteins (WITs), and WITs interact with myosin XI-i (Zhou et al., 2012; Tamura et al., 2013). The SUN-WIP-WIT complex is expected to have functions equivalent to the LINC complex in metazoans and fungi (Tamura et al., 2013). WIPs were biochemically identified as WPP-interacting factors (Xu et al., 2007a), and WITs were biochemically identified as WIP-interacting factors (Zhao et al., 2008). The C-terminal tail of WIP1, which is expected to be located in the perinuclear space and to interact with SUNs, has a low degree of similarity to the Opisthokont KASH domains (Zhou et al., 2012). The triple mutant *wip1wip2wip3* (Zhou et al., 2012) and the double mutant *wit1wit2* (Tamura et al., 2013) have spherical nuclei in the cell types that have spindle-shaped nuclei in WT plants. Myosin XI-i, whose anchoring to the nuclear membrane requires WITs, was identified as the gene responsible for *the kaku1* mutation (Tamura et al., 2013). Nuclei in *kaku1* are spherical in cells in which WT nuclei are spindle-shaped when visualized with nuclear-localized GFP (Nup50a-GFP; Tamura et al., 2013). Interestingly, it was observed that the NEs of *kaku1* are irregularly and intricately invaginated under the stable expression of SUN2-TagRFP or TEM (Tamura et al., 2013). Another putative *Arabidopsis* KASH domain protein, TIK (TIR-KASH protein), plays a role in nuclear morphology, specifically nuclear size (Graumann et al., 2014). SINEs (SUN-interacting NE proteins) have been identified as other components of plant LINC complexes (Zhou et al., 2014). *Arabidopsis* SINE1 is required for proper nuclear positioning in guard cells (Zhou et al., 2014). The *Z. mays* SINE1 homolog MLKS2 interacts with ZmSUN2 and is able to rescue the nuclear phenotype in the *Arabidopsis wip1 wip2 wip3* mutant (Gumber et al., 2019b). These results suggest that *the Zea* SINE-family KASH protein can be a substitute for *Arabidopsis* WIP-family KASH proteins for nuclear shape control (Gumber et al., 2019b).

#### Nucleoporins: *nup136/nup1* and *nup160*

The NPC comprises ~30 nucleoporins and is well characterized in vertebrates and yeast (D’angelo and Hetzer, 2008; Hoelz et al., 2011; Grossman et al., 2012). Interactive proteomics has identified 30 nucleoporins in *Arabidopsis* (Tamura et al., 2010). Nuclei in *nup136/nup1* (Tamura et al., 2010) and *nup160* (Parry, 2014) are more spherical than those in WT plants. *Arabidopsis* Nup136 is thought to be a functional homolog of vertebrate Nup153, which is physiologically associated with the nuclear lamina (Tamura et al., 2010; Tamura and Hara-Nishimura, 2011). As in the case of vertebrate Nup153, *Arabidopsis* Nup136 is a possible key determinant of nuclear structure through the interaction between NPCs and lamin-like structures (Tamura et al., 2010; Tamura and Hara-Nishimura, 2011).

### NE-Localized Proteins That Are Involved in the Deformation of the Nucleus/NE in Plants

#### NE Proteins of Which Overexpression Causes Deformation of the Nucleus/NE: KAKU4, CRWNs, and Nup136/Nup1

Among NE proteins, *KAKU4* (Goto et al., 2014), *CRWN4* (Sakamoto and Takagi, 2013), and *Nup136* (Tamura and Hara-Nishimura, 2011) cause deformations of the nucleus/NE. *KAKU4-GFP* overexpression causes nuclear membrane growth and NE deformation in whole seedlings of *Arabidopsis* and leaves of *N. tabacum* and *N. benthamiana* (Goto et al., 2014). Indeed, care is required to interpret the phenotypes caused by the overexpression of GFP-fused proteins, as they can impose artificial effects (Groves et al., 2019). However, *KAKU4* fused to another tag, *FLAG*, also causes NE deformation (Goto et al., 2014). The shape of VN in WT mature pollen grains, in which *KAKU4* is highly expressed, is irregular and reminiscent of the deformed shape of the nucleus in *KAKU4*-overexpressing cells, supporting the hypothesis that *KAKU4* affects NE shape (Goto et al., 2020). Co-overexpression of *FLAG-CRWN1* with *GFP-KAKU4* has a greater impact on NE shape in *N. tabacum* leaves (Goto et al., 2020). *CRWN4-GFP* overexpression causes nuclear elongation (Sakamoto and Takagi, 2013). Transgenic plants overexpressing Nup136-GFP have elongated nuclei in guard cells, leaf epidermal cells, and trichomes (Tamura and Hara-Nishimura, 2011).

#### NE-Localized Proteins of Which Deficiency Causes Deformed Nuclei: GIPs and Nup88

Some cytoskeleton-associated proteins also affect the shape of the nucleus/NE. One is the MT-associated protein GIP (Batzenschlager et al., 2014), and the other is a putative actin filament-associated protein, myosin XI-i (Tamura et al., 2013). Perinuclear MTs are nucleated from γ-tubulin complexes (γ-TuCs) located on the surface of the nucleus. The γ-TuC protein 3 (GCP3)-interacting protein 1 (GIP1), a γ-TuC component, was first discovered by a yeast two-hybrid screen (Janski et al., 2008). In *Arabidopsis*, there is a *GIP1* homologous gene, *GIP2*, which is collectively called *GIPs* (Janski et al., 2012). At the electron microscopy resolution, GIPs are localized on both sides of the NE (Batzenschlager et al., 2013). Nuclei of *gip1gip2* mutant cells exhibit an increased size and are highly deformed in root tips (Batzenschlager et al., 2013). The *gip1gip2* nuclei detected by DAPI staining are irregularly shaped and highly deformed (Batzenschlager et al., 2013), whereas the *kaku1/myosin XI-i* nuclei detected by Hoechst staining are spherical and hardly deformed, although the NE of *kaku1/myosin XI-i* is extensively invaginated (Tamura et al., 2013). It has also been shown that the *gip1gip2* mutant exhibits constitutive hyperosmotic stress response with stiffer and deformed nuclei (Goswami et al., 2020). In *N. benthamiana* plants, RNA silencing of *Nup88* causes shrunken nuclei in leaf cells (Ohtsu et al., 2014).

### Other Possible Factors That Can Be Involved in Nuclear Shape Determination

Although chromatin involvement seems to have relatively little effect on nuclear shape through animals and plants, it is reported that nuclei of BY-2 cells are elongated on long-term treatment with aphidicolin, an inhibitor of DNA polymerase (Yasuhara and Kitamoto, 2014). Recently, it is reported that hyperosmotic stress by 0.3 M mannitol treatment affects the nuclear shape in *Arabidopsis* root tips (Goswami et al., 2020). In yeast and metazoans, lipid biosynthesis and vesicle trafficking affect the shape of the nucleus/NE (Webster et al., 2009; Walters et al., 2012). Although *Arabidopsis* has genes *PAH1* and *PAH2* that are homologous to yeast *PAH1*, which is involved in lipid biosynthesis and affects the nuclear shape, unlike the yeast pah1-delta mutant, the volume of the nucleus was not greatly enlarged in cells of *pah1pah2-1* leaves, and its shape was not distorted (Eastmond et al., 2010). In *Scenedesmus acutus*, significant NE deformation and ONM budding were observed after treatment of brefeldin A, which is an inhibitor of vesicle transport (Noguchi et al., 1998). This implies that direct and/or indirect involvement of membrane traffic factors in the NE shape.

### Regulation of the Nuclear Shape by GIP, SUN-WIP-WIT-Myosin XI-i, and CRWN-KAKU4

In *Arabidopsis*, the nucleus changes from spherical shape to spindle shape at the same time as it grows in volume. Many reports mentioned above suggested that proteins localized in the NE, including cytoskeleton-related factors, contribute to the morphogenesis and maintenance of the nucleus and the NE. On the NE of *Arabidopsis*, the SUN-WIP-WIT complex connects the CRWN1-KAKU4 complex expected to form nuclear lamina with the cytoplasmic myosin XI-i, which is expected to be associated with the actin cytoskeleton (Figure 3). The intricate NE in the *kaku1*/*myosin XI-i* mutant suggests that the myosin pulls the NE under normal conditions (Tamura et al., 2013), which is consistent with the indication that the actin cytoskeleton pulls nuclei in metazoans (Mazumder and Shivashankar, 2010; Hampoelz and Lecuit, 2011). In contrast to the actin cytoskeleton, it is indicated that MTs exert compressive forces on the nucleus in metazoans (Mazumder and Shivashankar, 2010; Hampoelz and Lecuit, 2011), which may be related to that only *gip1gip2* shows a different phenotype on the nuclear shape compared to other mutants, including *kaku1/myosin XI-i* (Batzenschlager et al., 2013). As nuclei under hyperosmotic stress or those in *gip1gip2* shrink and become stiffer, nuclear shape and stiffness of the nucleus could be related (Goswami et al., 2020). The phenotype on the nuclear shape of the mutant lines defective in nuclear morphogenesis, except for *gip1gip2*, is similar in that nuclei are spherical in the cells in which nuclei are spindle-shaped in WT plants. However, the shape of the NE is different among mutant lines. The NE morphology of *crwn1-1* is smooth, different from the invaginated nuclei of *sun1-KO sun2-KD* and *wit2-1* (Zhou et al., 2015a). In addition, loss of *CRWN1* does not affect the localization of the SUN-WIP-WIT2 complex, suggesting that CRWN1 acts independently of the SUN-WIP-WIT complex (Zhou et al., 2015a). CRWN1 has a synergistic effect on NE deformation when coexpressed with *KAKU4* (Goto et al., 2014). Thus, CRWN1 may have the function of nuclear membrane growth and the NE deformation as *KAKU4* does. The NE shape in *KAKU4*-overexpressing cells is reminiscent of the NE shape in *Drosophila kugelkern*-overexpressing cells (Brandt et al., 2006; Polychronidou et al., 2010). *Drosophila kugelkern* is the INM protein required for nuclear elongation during cellularization in *Drosophila* embryo and induces nuclear membrane growth (Brandt et al., 2006). About *Drosophila kugelkern*, a model has been established in which the farnesylated C-terminus affects the nuclear shape by directly interacting with the lipid bilayer (Polychronidou and Großhans, 2011). In contrast, KAKU4 and CRWN1 have no predicted site for lipid modification, and the mechanism by which they change the shape of nuclear membranes is unclear. In the metazoans and yeasts, nucleoskeleton-cytoskeleton interactions, chromatin conformational changes, and altered lipid biosynthesis are involved in shaping the nuclear membrane (Polychronidou and Großhans, 2011). In *Arabidopsis*, at least nucleoskeleton (CRWNs and KAKU4) and cytoskeleton (myosin XI-i and GIP) near the NE seem to play a major role in regulating the nuclear shape (Tamura et al., 2015; Meier et al., 2016, 2017; Evans et al., 2020).

## Physiological Significance of Controlling the Nuclear Shape

### Overview of the Effects of the Nuclear Shape on Plant Physiology

There are two phenomena in which nuclear shape itself may be deeply involved: control of the order of migration of VN and SCs in pollen tubes (section “Migration of the Nucleus in Pollen and Roots”) and gene expression regulation (section “Regulation of Gene Expression”). In *Arabidopsis*, the absence of nuclear morphogenesis does not appear to be a fatal defect because plant growth is normal in mutants with aberrant nuclear shapes, such as *crwn1* (Wang et al., 2013) and *kaku4* (Goto et al., 2014). On the other hand, some nuclear shape mutants have defects in migration of the nucleus, maintenance of chromatin structure, meiosis, immunity, salicylic acid response, Ca^2+^ and reactive oxygen species (ROS) signaling, pollen development, and survival (section “Other Plant Phenotypes Affected by NE Proteins Involved in the Control of the Nuclear Shape”). Little is known about the direct effects of the NE deformation, including the formation of NR, on plant physiology, but studies in other organisms may be useful for the future development of plant research (section “Physiological Significance of NE Deformation”).

### Migration of the Nucleus in Pollen and Roots

When moving through the pollen tube, the VN precedes SCs. Time-lapse imaging of *in vitro* elongated pollen tubes has shown that the VN precedes sperm cells when they enter the pollen tube from pollen grains, and the migration order is mostly maintained during their migration in pollen tubes (Zhou and Meier, 2014), which implies that the positions of the VN and sperm cells when they enter the pollen tube are important in determining the migration order. The VN within pollen grains is irregularly shaped throughout various plant species, and the VN envelope is invaginated. Based on cases where the VN precedes and cases where SCs precede appear randomly in pollen tubes of *kaku4* of which mature pollen grains have the VN with less invaginated NE (Goto et al., 2020), the irregular shape of VNs is expected to help the VN slides into the pollen tube before the membrane-enclosed mass of sperm cells enter the pollen tube. Mutations in the genes encoding of NE proteins WIPs, WITs, or SUNs reverse the order of the migration of VN and SCs (Zhou and Meier, 2014; Zhou et al., 2015b). Recently, it became apparent that *wit1 wit2* VNs are shorter and more circular than WT VNs (Moser et al., 2020), which supports that the VN shape contributes to the control of the migration order of the VN and SCs. The possibility that nuclear mobility is more important than nuclear shape to determine the migration order of VN and SCs cannot be excluded, although *kaku1-4*, a myosin XI-i null mutant, exhibits a normal migration order (Zhou and Meier, 2014). The correct migration order of VN and SCs and/or keeping VNs at a certain distance from pollen tubes seems to be necessary for maintaining fertilization efficiency (Goto et al., 2014), Ca^2+^ and ROS signaling (Moser et al., 2020), pollen tube reception and seed production (Zhou and Meier, 2014; Zhou et al., 2015b).

In roots, the nucleus moves actively while changing its shape, suggesting that nuclear shape and nuclear movement can be linked (Chytilova et al., 2000). The *kaku1/myosin XI-i* with spherical nuclei moves much more slowly than WT spindle-shaped nuclei (Tamura et al., 2013). The flexibility of the nucleus to change its shape may affect the migration of the nucleus and the cell that harbors the nucleus. To be consistent with this idea, the neutrophils that migrate through tissues in mammalian cells have multilobed nuclei, typically exhibiting three or four lobes connected by thin DNA-containing filaments (Webster et al., 2009). Cells with less lobulated nuclei derived from the Pelger-Huet anomaly show a lower migration rate than normal control (Park et al., 1977).

### Regulation of Gene Expression

When mammalian cells are placed in small holes of various sizes and artificially changed their shapes, the nuclear shape also changes (Thomas et al., 2002). In this experimental system, collagen I synthesis correlates directly with the cell shape and nuclear shape index (Thomas et al., 2002). This report raises the possibility that the nuclear shape itself affects gene expression. Reduction of the chromocenter number and induction of expression of the gene encoding a linker histone (H1.3) are found in the organ-meristem boundary of *Arabidopsis*, in which the nuclear shape changes (Fal et al., 2021). In addition, NE proteins GIPs and CRWNs affect both the nuclear shape (section “NE-Localized Proteins That Are Involved in the Deformation of the Nucleus/NE in Plants”) and gene expression (as below). Transcriptomic analysis of *gip1gip2* compared to WT plants shows that 2092 genes containing many touch response genes are upregulated in *gip1gip2* (Goswami et al., 2020). Altered transcriptional profiles are detected in three single mutants *crwn1*, *crwn2*, and *crwn4* (Choi et al., 2019) and two double mutants *crwn1crwn2* (van Zanten et al., 2011; Choi et al., 2019) and *crwn1crwn4* (Choi et al., 2019; Sakamoto et al., 2020). More than 2000 differentially expressed genes were identified in the *crwn1crwn4* double mutant (Sakamoto et al., 2020). It is indicated that CRWNs could tether the locus of copper-associated genes to the nuclear periphery (Sakamoto et al., 2020), which suggests that gene expressions are regulated by tethering chromatin to the NE rather than a change in the nuclear shape. As GIP1 localizes not only the cytoplasmic side but also the nucleoplasmic side of the NE and CRWN1 localizes at the nucleoplasmic side of the NE, it is possible that they affect gene expression through binding of the proteins to the chromatin independently of the control of the nuclear shape.

In metazoans, it is well known that the tethering of chromatin to the NE mediates gene regulation (Czapiewski et al., 2016). Also in plants, it is suggested that association with the nuclear periphery is involved in the regulation of gene expression. Smith et al. showed that tethering of a reporter gene with nucleoporins affects the level of its expression in *Arabidopsis* (Smith et al., 2015). This suggests that plant NE proteins influence transcriptional activity by tethering genes to the nuclear periphery. It is shown that 10–20% of the regions on the chromosome arms are anchored at the nuclear periphery in plants (Bi et al., 2017). In *Arabidopsis*, CRWN1 is a key component of the lamina-chromatin network (Hu et al., 2019). CRWN1 suppresses the expression of the *PATHOGENESIS-RELATED1* (*PR1*) gene by enhancing the binding of NAC WITH TRANSMEMBRANE MOTIF1-LIKE9 (NTL9), an NAC transcription factor involved in plant immunity, to the *PR1* promoter (Guo et al., 2017). The *crwn1crwn2* double mutant shows enhanced resistance against the virulent pathogen (Guo et al., 2017). Loss of CRWN proteins induces the expression of the salicylic acid biosynthetic gene *ISOCHORISMATE SYNTHASE1*, which leads to spontaneous defense responses in *crwn1crwn2* and *crwn1crwn4* mutants (Choi et al., 2019). In addition to CRWNs, other NE proteins called NE-associated proteins (NEAPs) may connect to chromatin as NEAPs interact with a putative transcription factor called AtbZIP18 (Pawar et al., 2016). Tethering chromatin to the NE is associated with chromatin structure as well as the regulation of gene expression. DNA density is increased *crwn1crwn2* (Dittmer et al., 2007), and the chromocenter organization is altered in *crwn1crwn2* and *crwn4* (Wang et al., 2013). The absence of SUN1 and SUN2 proteins leads to a delay in meiotic progression and defects in synapsis and recombination (Varas et al., 2015). In *wifi*, the quintuple mutants deficient of *WIPs* and *WITs*, and *sun1sun4sun5*, chromocenters are further decondensed compared to WT (Poulet et al., 2017a). The absence of MLKS2 leads to multiple meiotic defects in *Z. mays*. The *Arabidopsis gip1gip2* mutants exhibit centromeric cohesion defects (Batzenschlager et al., 2015).

### Other Plant Phenotypes Affected by NE Proteins Involved in the Control of the Nuclear Shape

Some mutant lines that are deficient in genes affecting the nuclear shape show defects in seed germination, plant growth, survival, immune response, reproduction, and signal transduction. Whereas *crwn1crwn2* shows similar dormancy levels to WT (van Zanten et al., 2011), *crwn1crwn3* shows a low germination rate and hypersensitiveness to abscisic acid (Zhao et al., 2016). Whereas *crwn* single mutant lines have rosette leaves with a size similar to that of WT, some *crwn* double and triple mutant lines have smaller rosette leaves, and a mutant combining alleles in all four *CRWN* genes cannot be isolated (Wang et al., 2013). The *crwn1crwn2* double mutant shows enhanced resistance against the virulent pathogen as mentioned above (section “Regulation of Gene Expression”). Seed production is reduced in *wip1wip2wip3*, *wit1wit2*, and *wifi* (Zhou and Meier, 2014). It is shown that loss of *WITs* or *WIPs* impairs the pollen tube reception and SC-to-ovule migration (Zhou and Meier, 2014). *wit1wit2* and *wifi* pollen tubes are hyposensitive to exogenous hydrogen peroxide (H_2_O_2_), which induce pollen tube (Moser et al., 2020). Nuclear Ca^2+^ peaks observed after ROS (H_2_O_2_) treatment in growing pollen tubes are disrupted in the *wit1wit2* mutant (Moser et al., 2020). *nup136* mutants exhibited an early flowering phenotype (Tamura et al., 2010). NUP1 (also known as NUP136) deficiency affects both male and female gametophyte development, resulting in reduced seed production (Bao et al., 2019). In addition to the plant phenotype of *Arabidopsis* mutants above, the *Z. mays mlks2* mutants have defects in male meiosis, pollen viability, and stomatal complex development (Gumber et al., 2019b).

### Physiological Significance of NE Deformation

What is the physiological significance of NE deformation, including NR formation? NR is the intranuclear network formed by invaginations of the NE (section “NE Shapes Under Normal State”; Malhas et al., 2011). It has been suggested based on metazoan researches that the presence of an NR increases the surface-to-volume ratio within the nucleus, thereby facilitating both the entry and exit of Ca^2+^ (Bootman et al., 2009). It is also discussed that the cytoplasmic core of NR might also facilitate the translation of mRNA generated adjacent to the invagination, with NPCs within it permitting the prompt translocation to the cytoplasm of the NR core (Malhas et al., 2011). The recent findings of Ca^2+^ fluctuations in the nucleus in *Arabidopsis* pollen tubes, which are altered in *wit1wit2* (Moser et al., 2020), might be a clue to elucidate the relation between NR and Ca^2+^ signaling in plants. It is common between metazoans and plants that the invaginated NE often associates with nucleoli, which might facilitate mRNA export to the cytoplasm (Collings et al., 2000).

Nuclear egress of herpesviruses in vertebrates (Mettenleiter et al., 2006) and NE budding for ribonucleoprotein particle export during synaptic Wnt signaling in *Drosophila* (Speese et al., 2012) are accompanied by small and local NE deformation. Although similar changes in the NE shape during viral infections are also observed in plants (Malhas et al., 2011; mentioned in section “Changes in the NE Shape by a Viral Infection”), little is known about the mechanism underlying NE budding in plants. Nucleophagy (Kvam and Goldfarb, 2007; Park et al., 2009; Nezis et al., 2010) and NE rupture (Shah et al., 2017), both accompanied by a change in the NE shape, do not seem to have been elucidated yet in plants.

## Conclusion and Perspectives

Over the last two decades, progress has been made in the study of plant nuclear morphology. NE proteins that connect the cytoplasm to the nucleoplasm have been revealed one after another (summarized by Meier et al., 2017), and many of them are involved in the control of the nuclear shape and size in *A. thaliana*. Some mutants with an aberrant nuclear shape normally survive, whereas other mutants have defects in nuclear movement, control of migration order of VN and SCs in pollen tubes, maintenance of chromatin structure, meiosis, regulation of gene expression, plant immunity, salicylic acid response, Ca^2+^ and ROS signaling, pollen development, pollen tube reception, and seed production (Evans et al., 2020; Groves et al., 2020). Little is known about how the shape of the NE, including NR, is regulated. Now that many plant NE proteins have been identified, it could be expected that the molecular mechanisms underlying changes in the NE shape and new physiological phenomena accompanied by changes in the NE shape will be revealed.

## Author Contributions

All authors listed have made a substantial, direct and intellectual contribution to the work, and approved it for publication.

### Conflict of Interest

The authors declare that the research was conducted in the absence of any commercial or financial relationships that could be construed as a potential conflict of interest.
